# Chronic Marijuana Consumption Leading to High-Grade Atrioventricular Block in a Young Male

**DOI:** 10.7759/cureus.16202

**Published:** 2021-07-06

**Authors:** Amit Malviya, Shakeel A Khan, Anunay Gupta, Animesh Mishra

**Affiliations:** 1 Cardiology, North Eastern Indira Gandhi Regional Institute of Health and Medical Sciences, Shillong, IND; 2 Cardiology, Vardhman Mahavir Medical College and Safdarjung Hospital, New Delhi, IND

**Keywords:** cannabis, toxicity, electrophysiologic study, mechanism, high-grade atrioventricular block

## Abstract

Cannabis usage is increasing throughout the world for both medicinal and recreational purposes. Several countries and states have legalized cannabis, and physicians can expect to encounter more patients who use or abuse cannabis. Adverse cardiovascular effects of cannabis like myocardial infarction, cardiomyopathy, and arrhythmias have been well described but bradyarrhythmia is rare and the mechanisms are not well pronounced. A 26-year-old male with a history of chronic cannabis smoking presented with complaints of dizziness and recurrent syncope. The heart rate at presentation was 42 beats per minute and the rest of the physical examination was unremarkable. There was an atrioventricular (AV) block in the ECG and a subsequent electrophysiological study (EPS) showed a high-grade supra-Hisian (nodal) AV block with prolonged His-ventricular (HV) interval. The urinary screen was positive for tetrahydrocannabinol metabolite (11-Nor-9-carboxy THC). After ruling out other possible causes, a diagnosis of high-grade AV block due to chronic cannabis use was made. A dual-chamber pacemaker was implanted and the patient was discharged in stable condition. The arrhythmia did not improve completely at the three-month follow-up. We report a novel finding in cannabis-induced bradyarrhythmia. High-grade AV block with the electrophysiologic determination of the site of conduction blockade has not been reported previously. The mechanism of bradyarrhythmia is thought to be mediated by increased vagal tone. However, prolonged HV interval and persistent nature of block indicate that direct toxic effects of cannabis, through cannabinoid receptors 1 (CB1R), on the cardiac conduction system cannot be ruled out. Also, the possibility of cannabis arteritis involving microvasculature should be kept.

## Introduction

Cannabis is globally the most commonly used psychoactive substance and its use is increasing all over the world for both medicinal and recreational purposes. Physicians can expect to encounter more patients who use or abuse cannabis. Both acute and chronic adverse effects of cannabis on the cardiovascular system like myocardial infarction, cardiomyopathy, tachyarrhythmias, stroke, and cardiac arrest have been described, and the consumption of cannabis in various forms is a risk factor for cardiovascular disease in young adults [1]. The psychoactive constituent of cannabis, Δ9-tetrahydrocannabinol (THC), is an agonist of both cannabinoid receptors 1 and 2 (CB1R and CB2R) and exerts its psychoactive and adverse cardiovascular effects through the activation of CB1R in the central nervous and cardiovascular systems. There are several proposed mechanisms for cannabis-induced tachyarrhythmia, but it is not clear how it causes bradyarrhythmia and is primarily thought to be mediated by increased vagal tone [2]. Although, some cases of bradyarrhythmia have been described previously [3-13], high-grade atrioventricular (AV) block with electrophysiologic determination of the site of conduction blockade has not been reported to the best of our knowledge.

Our patient presented with a high-grade AV block. An electrophysiological study (EPS) revealed that the site of the blockade was supra-Hisian (nodal). AV conduction did not improve completely at follow-up despite complete abstinence, which indicates that cannabis-induced bradyarrhythmia is vagally mediated but direct damage to conduction fibers with persistent conduction defects is a possible mechanism as well.

## Case presentation

**Figure 1:**
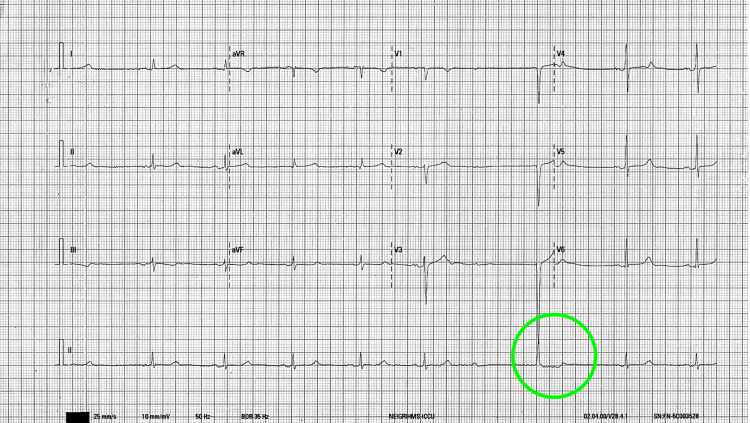
Sinus rhythm with a rate of 60 beats per minute followed by a sudden block of P wave and narrow escape (junctional).

**Figure 2:**
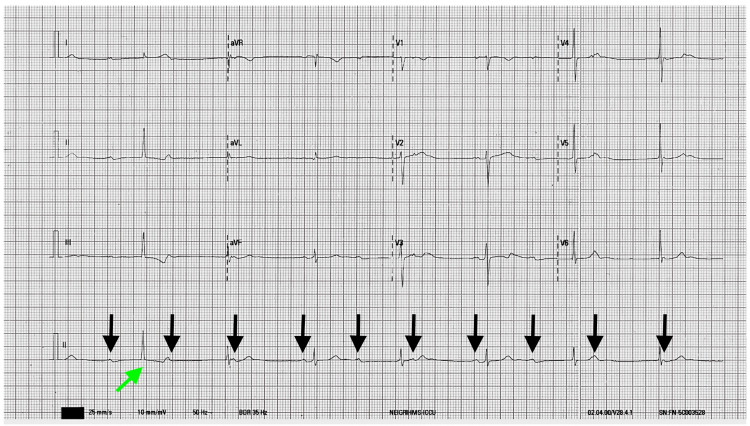
ECG shows complete heart block with AV dissociation and junctional escape rhythm with ventriculophasic arrhythmia (PP interval with QRS in between is narrower). Black arrows show P waves. Green arrow shows the escape beat from a different focus. AV: atrioventricular

A 26-year-old otherwise healthy male presented to emergency with complaints of dizziness and recurrent syncope. At presentation, his pulse was 42/min, blood pressure: 100/70 mmHg, temperature: 98.1°F, and respiratory rate: 16/min. The rest of the physical examination was unremarkable. ECG on presentation showed a high-grade AV block along with sinus node dysfunction (Figures 1-2).

There was no improvement in the heart rate by repeated administration of atropine (total up to 3 mg). The patient admitted to using cannabis for the last four years, and in the preceding two years, he was smoking 5-8 joints (4-5 g) daily. Prior ECG was within normal limits; therefore, ruling out congenital heart block. There was no family history of any heart disease. The blood counts and metabolic panel were within the normal limit. Serological tests for syphilis, Lyme disease, hepatitis B, hepatitis C, and HIV were negative. Serial troponin-I reports did not show any elevation and the patient’s thyroid-stimulating hormone was normal. Urinary screen 11-Nor-9-carboxy THC was positive (28 ng/ml; cut-off limit: 15 ng/ml). The other biochemistry values are presented in Table 1. 

**Table 1:** Routine hemogram and biochemistry values.

HEMOGRAM	
Hemoglobin	14.9 g/dl
Total leucocyte count	7000/microL
Neutrophil	69%
Lymphocyte	20%
Monocyte	7%
Eosinophil	4%
Platelet	2,50,000/microL
Erythrocyte sedimentation rate	45 mm/1st hour
BIOCHEMISTRY	
Urea	26 mg/dl
Serum creatinine	0.70 mg/dl
Serum magnesium	1.9 mg/dl
Serum sodium	143.1 mmol/L
Serum potassium	4.44 mmol/L
Serum chloride	111.8 mmol/L
Ionized calcium	1.02 mmol/L
Total serum calcium	1.99 mmol/L
Serum glucose	132 mg/dl
Creatine phosphokinase	106 U/L
Creatine kinase-MB	52 U/L
Total protein	7.4 g/dl
Albumin(A)	4.7 g/dl
Globulin(G)	2.7 g/dl
A/G ratio	1.7
Total bilirubin	0.9 mg/dl
Alanine aminotransferase	22 U/L
Aspartate transaminase	25 U/L
Alkaline phosphatase	69 U/L
Troponin-I	Negative (TnIA2=0.00 ng/ml)
Total cholesterol	154 mg/dl
High density lipoprotein	52 mg/dl
Low density lipoprotein	80 mg/dl
Triglyceride	102 mg/dl
FreeT4	0.9 ng/dl
FreeT3	3.20 pg/ml
Thyroid-stimulating hormone	3.9 uIU/ml
11-Nor-9-carboxy THC	28 ng/ml

**Figure 3:**
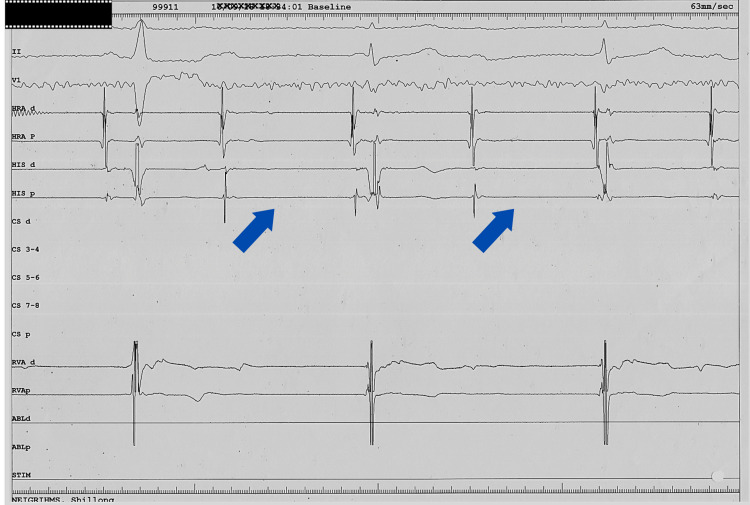
EPS shows high-grade AV block, only the first A is conducted with an LBBB, second and third QRS are junctional escape beats. Arrows show A not followed by H and V suggesting AV nodal block. EPS: electrophysiological study; AV: atrioventricular; LBBB: left bundle branch block

The chest X-ray was normal. There was no clinical/biochemical or radiological evidence to suggest rheumatic, sarcoid heart disease, or myocarditis. On 2D-echocardiography, cardiac chambers and valves were normal with normal left ventricular function (ejection fraction: 66%). The EPS showed a high-grade AV block with atrium to His bundle (AH) interval: 180 ms, His-ventricular (HV) interval: 85 ms, PP interval: 648 ms, RR interval: 1274 ms (Figure 3). Coronary angiography showed normal coronaries.

A dual-chamber pacemaker (Medtronic, model: ATDRL1, Medtronic Inc, Minneapolis, USA) was implanted and the patient was discharged in stable condition. At discharge, the rhythm was ApVp (atrial paced ventricular paced) and it was AsVp (atrial sensed ventricular paced) 70% of the time, and ApVp 30% of times at follow-up after three months. At follow-up, the patient admitted to completing abstinence from cannabis smoking.

## Discussion

Cannabis smoking is mostly associated with chronic sinus tachycardia and other tachyarrhythmias like atrial fibrillation and ventricular tachycardia. A scoping study of 27 cases of arrhythmia associated with cannabis reported that most cases were young males and the mortality rate was high with cannabis-associated arrhythmia (11%). Atrial fibrillation (26%) and ventricular fibrillation (22%) were the most common arrhythmias. Bradyarrhythmias were not common; first- and second-degree AV block and sinus arrest (3.7% each) were the common findings in the bradyarrhythmia group [14]. In another extensive review about the association of cannabis use and cardiac dysrhythmias, out of 42 subjects of bradyarrhythmia, third-degree AV block was found in 12% of the cases while symptomatic bradycardia was present in 10% of the cases [15]

Table 2 depicts the reported cases of severe bradyarrhythmia due to cannabis toxicity. Out of 11 cases of severe bradyarrhythmia reported to date, three cases were of third-degree heart block, two cases were of second-degree heart block, and six cases were of sinus arrest. Pacemaker implantation was done in two cases of complete heart block and three cases of sinus arrest, and in the rest of the five cases, conduction improved after abstinence from cannabis while one case was lost to follow-up. In none of the reported cases an EPS was done to determine the site of the blockade.

**Table 2:** Cases of severe bradyarrhythmia due to marijuana toxicity. AV: atrioventricular; TAPVC: total anomalous pulmonary venous connection

Sl.no	Patient characteristics	Presentation	Management	Reference
	A 46-year-old lady with a history of using 2 bags of marijuana daily for about 15 years	Presented with 2:1 block which further deteriorated to complete heart block with heart rate at presentation: 35 bpm	The patient underwent successful elective placement of a dual-chamber permanent pacemaker	Heeralall R et al. in 2006 [3]
	A 51-year-old lady who took 1-2 bags of marijuana 4-5 times a week for many years	Presented with intermittent 2:1 AV block and third-degree AV block with heart rate at presentation: 39 bpm	The patient underwent successful elective placement of a dual-chamber permanent pacemaker	Mithawala P et al. in 2019 [4]
	A 19-year-old man with a history of cannabis use	Presented with episodes of third-degree AV block with heart rate at presentation: 100 bpm	Isoprenaline infusion for 1 day, no further episode of AV block, aside from physiological Wenckebach phenomenon	Van Keer JM in 2019 [5]
	A 21-year-old Black male with a 2-year history of marijuana consumption	Presented with sinus bradycardia and intermittent second-degree AV block with heart rate at presentation: 36 bpm	AV block disappeared after abstinence from marijuana for 72 hours but sinus bradycardia persisted	Akins et al. in 1981 [6]
	ECG of 37 patients with positive urine screening for marijuana (median age of 15 years)	Two patients presented with first-degree AV block and one with second-degree AV block (Mobitz I) with heart rate at presentation: 51 bpm	In follow-up, patient continued the use of marijuana on a regular basis and his follow-up ECG demonstrated Mobitz type I second-degree AV block	Robinson JA et al. in 2017 [7]
	A 40-year-old male with a history of smoking marijuana occasionally	Presented with four episodes of near syncope related to strenuous exercise over a period of 6 months, on incremental treadmill exercise test (Bruce protocol). At 30 seconds of recovery, he became syncopal and was noted to have sinus arrest, followed by a 7-second period of ventricular asystole	The patient underwent successful elective placement of a dual-chamber permanent pacemaker	Dockery et al. in 2007 [8]
	A 21-year-old male operated during infancy for an unobstructed TAPVC with an excellent result, who used to smoke about 1 g of marijuana mixed with the tobacco of one cigarette per day	Presented with two episodes of loss of consciousness. On Holter, the underlying rhythm was sinus or sinus arrhythmia with a rate between 17 and 147 bpm with 17 pauses of greater than 2 seconds, the longest being 5.8 seconds	A repeat Holter was normal following a two-week cessation of marijuana usage and again when carried out 3 months later	Menahem in 2013 [9]
	A 28-year-old male with a history of marijuana use on occasions	Presented with loss of consciousness with a history of similar episodes after using marijuana. Telemetry revealed several sinus pauses	The patient underwent successful elective placement of a single chamber permanent pacemaker	Brancheau et al. in 2016 [10]
	A 54-year-old female with a history of smoking marijuana 3 g/day (joint) and regular cigarette 1 pack/day	Presented with intermittent episodes of pre-syncope following acute inhalation of marijuana. An episode of sinus arrest with a 4.6-second pause with heart rate of 38 bpm was noticed, another prolonged asystole after a few beats was observed	The patient advised for abstinence from marijuana and follow-up but the patient did not return	Grieve-Eglin et al. 2018 [11]
	A 17-year-old female with a history of smoking marijuana about 3-4 g/day for 1 month and a history of malnutrition secondary to anorexia nervosa	At the time of presentation, patient was under the acute influence of marijuana, showed marked sinus bradycardia with a heart rate of 44 bpm and junctional rhythm	After abstinence, patient’s ECGs revealed an improved heart rate, sinus rhythm, and resolution of T-wave inversion	Chaphekar et al. in 2019 [12]
	A 27-year-old male with a history of daily marijuana use	The patient presented with recurrent syncopal episodes. Findings of loop recorder were suggestive of sick sinus syndrome with sinus pause of more than 3 seconds	The patient underwent successful elective placement of a single chamber permanent pacemaker	Iqbal et al. in 2019 [13]

There are several proposed mechanisms for tachyarrhythmias caused due to cannabis, such as altered conduction property of myocardial tissue, cardiac ion channel modulation, autonomic dysfunction due to imbalance between sympathetic and parasympathetic outflow, type 2 myocardial infarction due to tachycardia, elevated carboxyhemoglobin, slow coronary flow due to endothelial dysfunction, coronary vasospasm, and increased platelet aggregation leading to ischemic injuries/scar creating a milieu for arrhythmia. However, the exact mechanism by which it causes bradyarrhythmia is not well known [1,2]. The agonistic action of THC on CB1R causes sympathetic inhibition and increased cardiac vagal tone leading to bradycardia. Acute cannabis smoking of low to moderate doses causes tachycardia in the first 60 minutes from sympathetic stimulation and parasympathetic inhibition, while large and/or chronic doses cause parasympathetic inhibition [16]. The EP effects of intravenous THC in human experimental studies on cardiac conduction include a change in P wave morphology, decrease in sinoatrial (SA) conduction, delay in AH interval, and decrease in AV node refractory period. However, in this case, the HV interval was prolonged, which is not explainable completely by previously described mechanisms as increased vagal tone is not known to affect the conduction system beyond the AV node. Therefore, it is possible that either cannabis has some direct toxic effects on cardiac conduction systems through cannabinoid receptors 1 or due to the involvement of microvasculature supplying the AV node due to cannabis arteritis [17]. Although cannabis arteritis is rare and mainly reported to involve peripheral vasculature, this may be kept as a possibility in the absence of any evidence of congenital heart block, connective tissue disease, autoimmune disease, myopathies, or infection.

**Figure 4:**
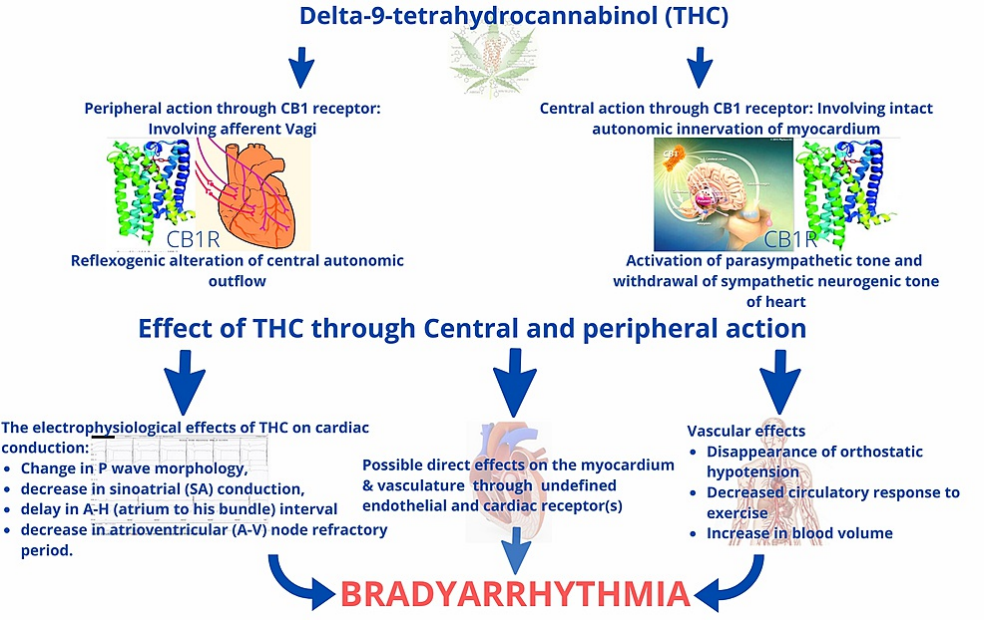
The mechanism of bradyarrhythmia caused by cannabis. AH: atrium to His bundle; AV: atrioventricular; CB1R: cannabinoid receptor 1; SA: sinoatrial; THC: delta-9-tetrahydrocannabinol Adapted from Cavero et al. [19] and Goyal et al. [20]

Upon application of the Naranjo scale for estimating the likelihood of an adverse drug reaction [18] for this case, the score was 2, which is suggestive of a possible adverse drug reaction. Figure 4 demonstrates the possible mechanism of bradycardia caused by cannabis [19,20]. Our patient consumed cannabis for about four years and his previous medical record was not suggestive of any risk factor for complete heart block except chronic cannabis use. He was implanted permanent pacemaker after two weeks of observation. During follow-up, the patient claimed complete abstinence from cannabis use but the AV conduction did not improve completely on pacemaker interrogation on three months of follow-up.

## Conclusions

Chronic use of cannabis causes a decrease in heart rate due to reduced sympathetic activity and increased parasympathetic activity. The EP effects of THC on cardiac conduction include a change in P wave morphology, decrease in SA conduction, delay in AH interval, and decrease in AV node refractory period. In this index case, we showed that cannabis can be linked to severe bradyarrhythmia, primarily mediated by the enhanced vagal tone and direct damage to the conduction system due to toxic effects, and these effects may be persistent in nature. Physicians should expect to encounter more patients who use or abuse marijuana, and awareness regarding such cardiovascular side effects of cannabis is warranted. Screening for cannabis use should be encouraged, especially in young patients presenting with arrhythmias.
